# Section on Dermatology

**Published:** 1896-03

**Authors:** 


### Section on Dermatology.

The following circular letter has been issued by the officers of the Section on Dermatology:

Galveston, Texas, February 15, 1896.

My Dear Doctor:—The section on Dermatology has not always received the attention which we think it merits. At the next meeting of the Association, in Fort Worth, we are very anxious to present as much valuable and instructive matter in this section as we can possibly collect. To this end, we would be very much pleased if you would contribute a paper or report a case, or cases, and if you can do so, we will be very much obliged if you will send us the title of your contribution by March 15th, so that it may appear upon the programme.

Very truly yours,

Geo. H. Lee, M. D.,
Chairman Section on Dermatology, T. S. M. A.

R. W. Knox, M. D., Secretary.

